# Workflow and Clinical Outcomes After Implementation of a Structured Trauma Protocol in a Resource-Constrained Emergency Department: A Single-Center Retrospective Before-and-After Cohort Study

**DOI:** 10.3390/healthcare14213588

**Published:** 2026-10-23

**Authors:** Reşad Beyoğlu, Oğuz Küçükyılmaz

**Affiliations:** Department of Emergency Medicine, Bandırma Training and Research Hospital, Faculty of Medicine, Bandırma Onyedi Eylül University, Bandırma 10200, Türkiye

**Keywords:** trauma care, emergency department, workflow optimization, point-of-care ultrasound, computed tomography, quality improvement, resource-constrained care, retrospective before-and-after study

## Abstract

**Highlights:**

**What are the main findings?**

- A three-component quality-improvement trauma workflow was associated with shorter CT access, consultation-request, and admission-decision times in a non-hybrid emergency department.
- The study was a retrospective before-and-after evaluation of routinely collected clinical records; clinical outcome findings are exploratory and should not be interpreted causally.

**What are the implications of the main findings?**

- Coordinating early bedside ultrasound, CT readiness, and brief team debriefing may support trauma workflow in settings without hybrid resuscitation-imaging infrastructure.
- The main signal of this study is operational; mortality and procedure findings require confirmation in controlled or multicenter implementation studies.

**Abstract:**

**Background/Objectives:** Coordinated imaging and clinical decision-making are central to trauma care, but delays may occur in emergency departments without hybrid resuscitation–imaging facilities. We examined associations between a structured departmental quality-improvement workflow and trauma-process times in a resource-constrained emergency department. **Methods:** We retrospectively compared 360 consecutive eligible trauma patients (180 before and 180 after implementation). The post-protocol workflow combined early extended focused assessment with sonography for trauma (eFAST), prioritized computed tomography (CT) coordination, and brief post-resuscitation debriefing. The primary outcome was CT access time. Other workflow outcomes were evaluated as secondary endpoints; procedural and mortality findings were exploratory. **Results:** Median CT access time was 22.38 min (IQR 16.02–28.75) before implementation and 10.98 min (IQR 7.53–14.71) afterward (*p* < 0.001); the mean difference was −11.10 min (95% CI −12.69 to −9.50). Median consultation-request time was 23.23 versus 13.69 min, and median admission-decision time was 125.95 versus 49.08 min (both *p* < 0.001). Pre-CT tube thoracostomy was recorded more often in the post-protocol phase (3.3% vs. 11.7%; RR 3.50, 95% CI 1.45–8.47; *p* = 0.003). Differences in mortality were exploratory and cannot be attributed to the protocol. **Conclusions:** The post-protocol phase was associated with shorter trauma workflow intervals, particularly CT access time. Because this retrospective, uncontrolled comparison followed calendar sequence, it cannot establish a causal protocol effect. Procedural and mortality findings are hypothesis-generating; controlled studies are needed to assess reproducibility and patient-centered benefit.

## 1. Introduction

Trauma resuscitation depends on rapid recognition of time-sensitive injury, coordinated imaging, and early procedural decision-making. Delays in computed tomography (CT), specialist consultation, operative intervention, and disposition can lengthen the interval to definitive care, particularly in emergency departments that do not have hybrid trauma bays or colocated CT scanners [1,2,3,4,5,6,7]. In these environments, improvement may depend less on new infrastructure than on how existing personnel, imaging resources, and communication pathways are organized.

Point-of-care ultrasonography has an established role in the initial assessment of injured patients and may support earlier recognition of pneumothorax, hemothorax, pericardial effusion, and intra-abdominal free fluid [1,3,8,9]. In parallel, coordinated CT activation and whole-body imaging strategies have been studied as approaches to reduce diagnostic delay in patients with major trauma [4,10,11,12,13]. Team behaviors and structured debriefing may also influence resuscitation performance by clarifying roles, identifying recurrent bottlenecks, and promoting iterative process improvement [14,15,16].

These interventions are often evaluated separately. Evidence is more limited for pragmatic bundles that combine bedside imaging, CT coordination, and team-based feedback in resource-constrained emergency departments. Such bundles are attractive because they can be implemented without construction of a hybrid emergency room or major increases in staffing, but their real-world effects may be difficult to separate from secular trends and learning effects [17,18].

We examined changes in trauma workflow and selected clinical outcomes across two calendar-sequenced phases surrounding implementation of a three-component quality-improvement protocol. CT access time was the primary outcome. Time to first-image availability, consultation-request time, and admission-decision time were secondary workflow outcomes. Procedural and mortality outcomes were exploratory because the before-and-after design was uncontrolled.

## 2. Materials and Methods

### 2.1. Study Design and Setting

We conducted a single-center retrospective before-and-after cohort study using routinely recorded clinical data from patients treated between 1 December 2024 and 1 December 2025 in the Emergency Department of Bandırma Training and Research Hospital, affiliated with Bandırma Onyedi Eylül University Faculty of Medicine, Türkiye. These dates describe the period of clinical care, not the period of research data extraction. The department does not have a hybrid resuscitation–CT suite. The workflow changes became part of routine departmental quality improvement independently of the subsequent research evaluation.

The trauma workflow was introduced for clinical quality improvement, not as a research intervention. Approximately six months after its introduction, the investigators decided to evaluate pre-implementation and post-implementation records retrospectively. No examination, treatment, or patient contact was undertaken specifically for this research; research activities began after ethics approval.

The pre-protocol and post-protocol phases each included 180 consecutive eligible patients, selected in calendar sequence rather than by random allocation. The ethics-approved research plan specified 360 patients. The de-identified analytic file retained case order but not encounter dates, so the exact phase-boundary date could not be reconstructed from that file. A distinct run-in interval was not documented. Research-specific record abstraction, dataset creation, and statistical analyses began after ethics approval. Reporting followed STROBE and SQUIRE 2.0 [19,20].

### 2.2. Participants

All consecutive patients who triggered trauma-team evaluation during the clinical data-coverage period were assessed for eligibility. Inclusion criteria were blunt or penetrating trauma requiring trauma-team activation and direct presentation without completed pre-arrival cross-sectional imaging. Exclusion criteria were isolated minor injuries not requiring comprehensive trauma assessment, dead-on-arrival patients, interfacility transfers with completed imaging, and missing or incomplete clinical records. The final analytic cohort comprised 360 patients (180 per phase). The original screening log was not retained in the de-identified analytic dataset; consequently, the numbers screened and excluded within each individual exclusion category could not be reconstructed reliably and were not estimated. The exact historical trauma-team activation thresholds were likewise not retained in the analytic file; therefore, phase-specific activation thresholds could not be independently re-verified during revision.

### 2.3. Structured Trauma Workflow Protocol

The post-protocol pathway consisted of three operational components implemented as a bundle. Operational definitions and the decision bundle are provided in Table S1, and the protocol pathway is shown in Figure S1. The intervention modified workflow organization rather than assigning patients to an experimental treatment strategy:

Early eFAST assessment. During the post-protocol phase, eFAST was incorporated into the initial resuscitation workflow. The available analytic file contained a thoracic eFAST result for all 180 post-protocol patients; structured pre-protocol eFAST documentation was not available, preventing reliable quantification of pre-protocol eFAST use. Examinations included pericardial, intraperitoneal, pleural, and lung views used in routine trauma assessment. Findings were incorporated into bedside clinical decision-making. The number of individual operators and formal credentialing records could not be reconstructed from the de-identified analytic file and are acknowledged as implementation-reporting limitations.

Prioritized CT coordination. CT personnel were notified at trauma-team activation or promptly after ambulance arrival. Scanner preparation and transfer arrangements proceeded in parallel with initial stabilization. CT pre-notification was documented in 168/180 (93.3%) post-protocol records versus 30/180 (16.7%) pre-protocol records. The workflow sought to shorten avoidable waiting and transfer intervals; it did not target CT acquisition or picture archiving and communication system (PACS) processing. Scanner configuration, radiographer staffing, reconstruction protocols, and contemporaneous PACS conditions were not recorded in the analytic dataset. Consequently, differences in scan-initiation-to-first-image time cannot be attributed specifically to pre-notification.

Brief post-resuscitation debriefing. After major resuscitations, the team leader conducted a brief structured review focused on delays, communication, role allocation, equipment or spatial constraints, and practical changes for subsequent cases. Documented debriefing was present in 146/180 (81.1%) post-protocol records compared with 19/180 (10.6%) pre-protocol records. The purpose was iterative workflow refinement rather than individual performance appraisal.

### 2.4. Data Collection and Definitions

Clinical and workflow variables were retrospectively extracted after ethics approval from routinely completed trauma documentation and electronic hospital records. Variables included age, sex, Glasgow Coma Scale (GCS), systolic blood pressure (SBP), respiratory rate (RR), heart rate, Injury Severity Score (ISS), shock index, mechanism and anatomical distribution of injury, eFAST findings, procedural interventions, blood product use, and mortality. During source-data reconciliation, the previously tabulated RTS values were found to be inconsistent with the recorded GCS, SBP, and RR. RTS was therefore recalculated for all patients using the standard weighted Revised Trauma Score: RTS = 0.9368 (GCS code) + 0.7326 (SBP code) + 0.2908 (RR code), with coded values defined according to the original RTS method [21]. Shock index was verified as heart rate divided by SBP. TRISS-predicted mortality was calculated descriptively using the recalculated RTS, ISS, age index, and blunt/penetrating mechanism using the original TRISS coefficients [22]. Anatomical injury categories were not mutually exclusive; patients with injuries involving more than one body region could contribute to multiple anatomical categories. Mechanism-of-injury categories were mutually exclusive.

CT access time was the interval from ED arrival to the start of CT imaging. Time to first-image availability was measured from scan initiation to availability of the first image in the electronic system. Consultation-request and admission-decision intervals were measured from ED arrival. All four prespecified workflow-time variables were available for all 360 patients in the final analytic cohort (360/360 for each variable). This high completeness applies to the selected analytic cohort and does not establish the absence of missing data among screened or excluded patients. An admission-decision interval was also available for all 26 patients recorded as ED deaths, but the original event labels and timestamps were unavailable to verify the temporal sequence for each case. Early tube thoracostomy was defined as thoracostomy before CT. ED mortality meant death during the ED episode; in-hospital mortality included deaths before discharge. Because original raw timestamps were not retained, the exact electronic source of the ED-arrival time (physical arrival or registration) could not be rechecked.

### 2.5. eFAST Diagnostic Assessment

When a reference standard was available, eFAST findings were compared with CT and/or operative findings. In the post-protocol phase, thoracic eFAST results were available for 180/180 patients; 31 records were positive and 149 negative. CT/operative confirmation fields were available for the same post-protocol records and matched the thoracic eFAST classification in this analytic dataset. For intra-abdominal free fluid, paired eFAST and reference-standard entries were retained for 33 post-protocol examinations; records without a paired abdominal index/reference result were not treated as negative tests and were excluded from the 2 × 2 diagnostic analysis. Diagnostic performance was summarized as sensitivity, specificity, positive predictive value (PPV), negative predictive value (NPV), and overall accuracy with Wilson 95% confidence intervals. Because structured pre-protocol eFAST fields were not available and reference-standard ascertainment was limited for the abdominal analysis, verification bias remains possible.

### 2.6. Outcomes

The primary outcome was CT access time, selected because the quality-improvement workflow targeted delays between ED arrival and CT initiation. It is a measurable process outcome that does not depend on assumptions about downstream clinical effects. Secondary workflow outcomes were time to first-image availability, consultation-request time, and admission-decision time. Tube thoracostomy before CT, blood product use, emergency surgery, ED mortality, in-hospital mortality, and descriptive eFAST accuracy were treated as exploratory clinical and diagnostic outcomes. No multiplicity adjustment was applied; the corresponding *p*-values do not establish treatment or survival benefit.

### 2.7. Statistical Analysis

Continuous variables were summarized as mean ± standard deviation (SD) and, for workflow-time outcomes, additionally as median and interquartile range (IQR). Normality was assessed using the Shapiro–Wilk test and homogeneity of variance for baseline t-test comparisons using Levene’s test; Welch’s *t*-test was prespecified if variance homogeneity was violated. Because at least one phase of the primary CT-access outcome departed from normality and because time variables may be right-skewed, between-phase comparisons of workflow times were performed using the Mann–Whitney U test; mean differences with 95% confidence intervals were retained to facilitate comparison with the original analysis. Baseline continuous variables were compared using independent-samples t tests, and categorical variables using Pearson chi-square tests or Fisher’s exact test for sparse cells. Binary clinical outcomes are reported as risk ratios (RRs) with 95% confidence intervals. No multiplicity adjustment was applied; therefore, secondary and exploratory *p*-values are interpreted cautiously.

Given the limited number of in-hospital deaths (39 events), the overlap between physiologic predictors, and the nonrandomized calendar-based phase allocation, the previously submitted five-predictor multivariable mortality model was not retained. Re-analysis of the uploaded patient-level values did not reproduce the originally reported adjusted phase estimate. Mortality is therefore presented descriptively using crude effect estimates and is interpreted as hypothesis-generating. No adjusted mortality estimate is presented in the Abstract, Highlights, Results, or Conclusions.

All tests were two-sided, and *p* < 0.05 was considered statistically significant. The original study analyses were performed using IBM SPSS Statistics, version 29 (IBM Corp., Armonk, NY, USA). Re-analysis was performed from the patient-level analytic dataset. RTS and TRISS calculations were recomputed from their component variables as described above. Month-by-month run-chart or interrupted-time-series analysis could not be performed because encounter dates were not retained in the de-identified analytic file used for this revision.

### 2.8. Ethics

The study was approved by the Bandırma Onyedi Eylül University Non-Interventional Research Ethics Committee (Approval No. E-67961857-050.04-2600006698; 13 January 2026). It was a retrospective, non-interventional analysis of de-identified clinical records from 1 December 2024 to 1 December 2025. The departmental trauma workflow had been introduced as part of routine clinical quality improvement independently of the subsequent research. Research-specific data extraction and analysis began only after ethics approval; no additional patient contact or study-specific intervention was undertaken.

## 3. Results

### 3.1. Cohort Characteristics

After patient-level re-analysis, mean age was 40.4 ± 15.3 years in the pre-protocol group and 42.5 ± 14.9 years in the post-protocol group (*p* = 0.187). Recalculated mean RTS was 7.39 ± 0.58 and 7.44 ± 0.52, respectively (*p* = 0.409). Mean ISS was 15.77 ± 7.97 versus 14.32 ± 7.27 (*p* = 0.073). Baseline characteristics and injury profile are summarized in Table 1. Detailed severity distributions are presented in Section 3.5.

### 3.2. Workflow Time Metrics

**Figure 1:**
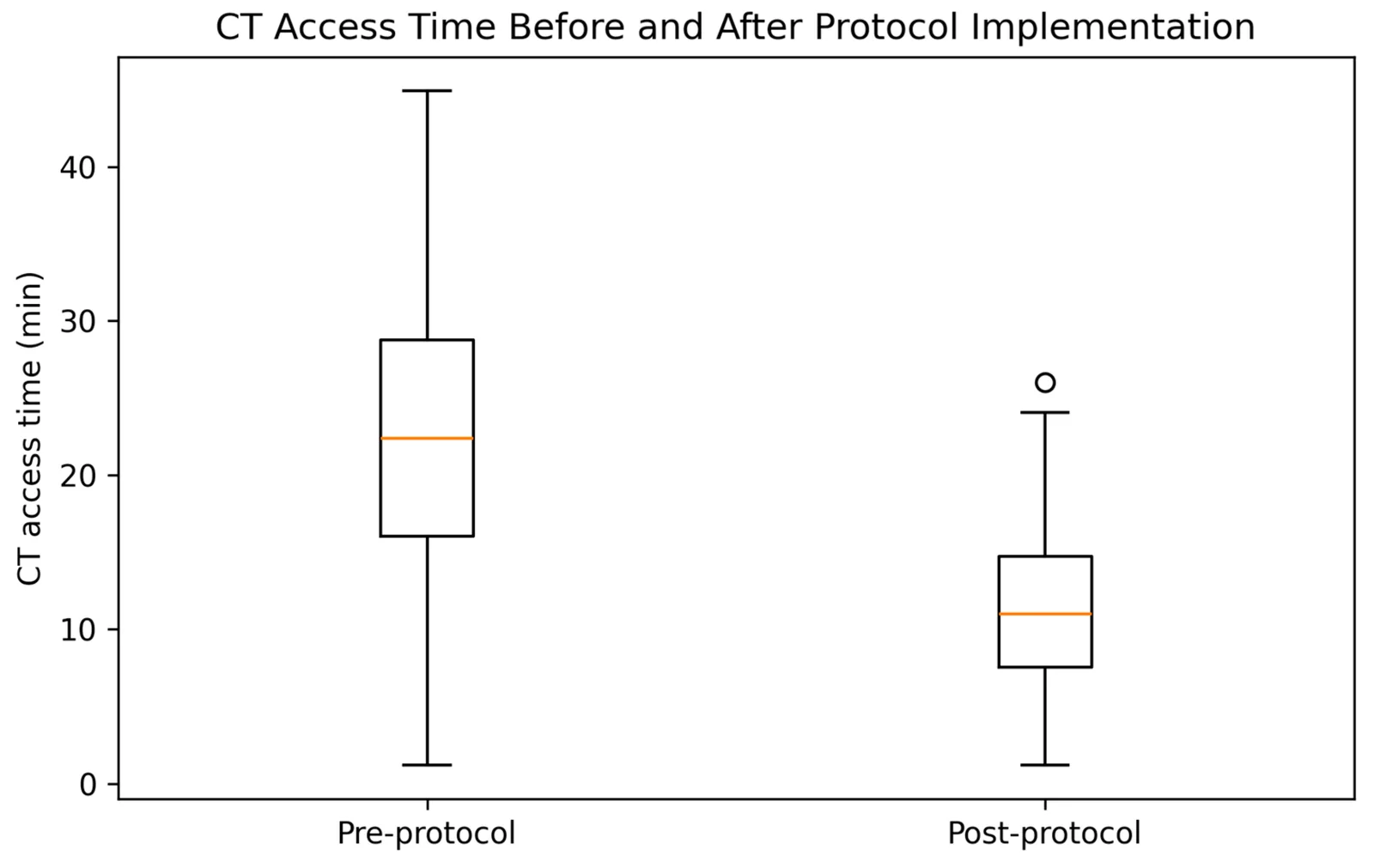
Distribution of CT access time before and after protocol implementation. Box plots show median, interquartile range, and individual outliers.

**Figure 2:**
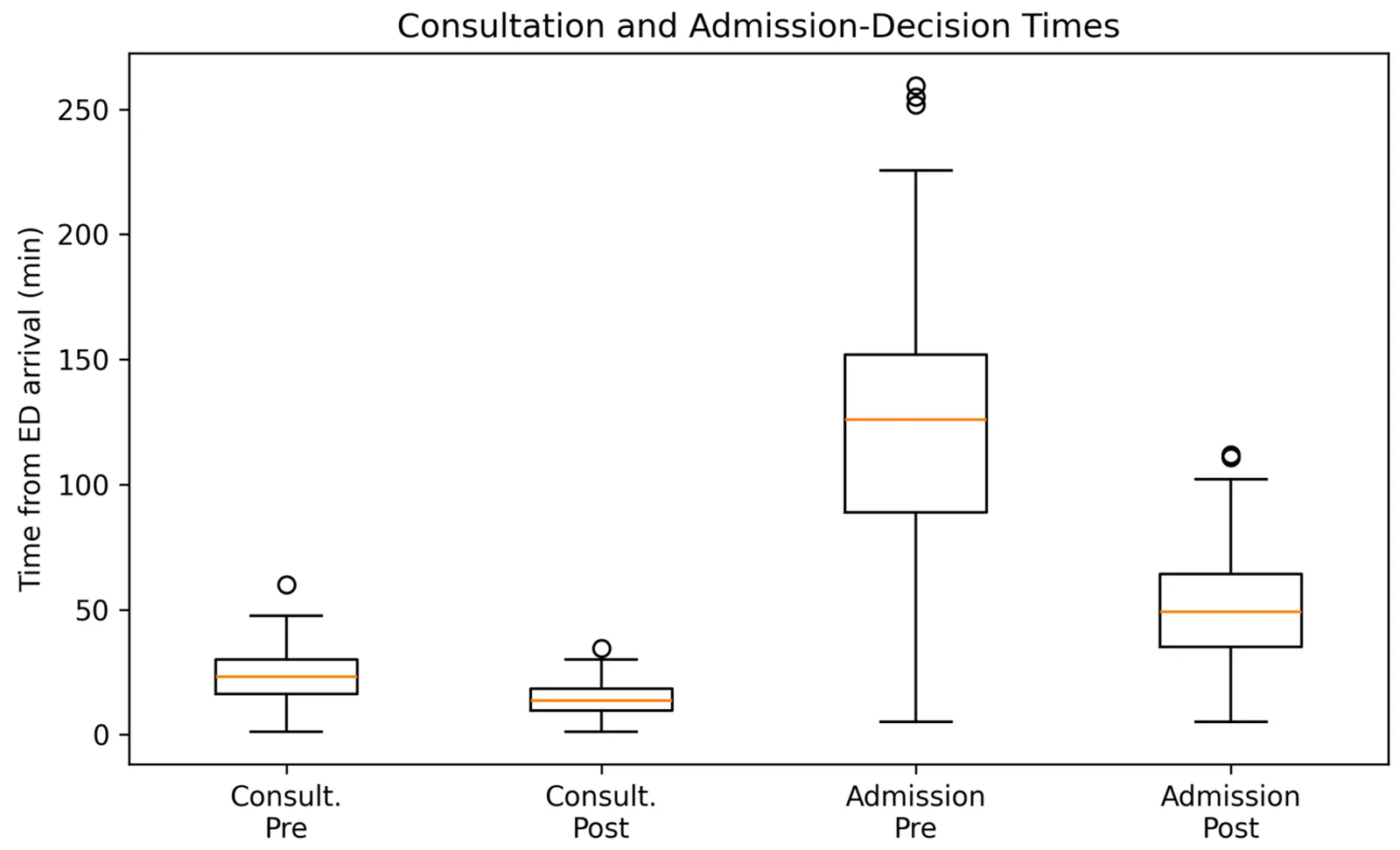
Distribution of consultation-request and admission-decision times before and after protocol implementation. Box plots show median, interquartile range, and individual outliers.

The post-protocol phase was associated with shorter workflow intervals (Table 2). Median CT access time decreased from 22.38 min (IQR 16.02–28.75) to 10.98 min (IQR 7.53–14.71; Mann–Whitney *p* < 0.001). Mean CT access time decreased from 22.42 ± 9.55 to 11.33 ± 5.14 min (mean difference −11.10 min, 95% CI −12.69 to −9.50). Median time to first image decreased from 8.15 to 4.01 min, consultation-request time from 23.23 to 13.69 min, and admission-decision time from 125.95 to 49.08 min (all *p* < 0.001). The analytic dataset contained values for each time outcome in all 180 patients per phase. The distributions of CT access time and consultation/admission-decision intervals are shown in Figure 1 and Figure 2, respectively.

### 3.3. eFAST Findings and Early Procedures

In the post-protocol phase, structured thoracic eFAST results were available for all 180 patients: 31 were positive and 149 negative. In the available analytic file, thoracic eFAST classification was concordant with the CT/operative confirmation field. Structured pre-protocol eFAST results were not retained, so phase-specific eFAST utilization before implementation could not be quantified. Abdominal eFAST had reference-standard data in 33 post-protocol examinations.

For intra-abdominal free fluid, eFAST yielded six true-positive, five false-positive, 19 true-negative, and three false-negative examinations (*n* = 33 with paired reference-standard data). Sensitivity was 66.7% (95% CI 35.4–87.9), specificity 79.2% (95% CI 59.5–90.8), PPV 54.5% (95% CI 28.0–78.7), NPV 86.4% (95% CI 66.7–95.3), and overall accuracy 75.8% (95% CI 59.0–87.2) (Table 3). In the five false-positive cases, the reference field was negative for intra-abdominal free fluid; two underwent emergency surgery, but the indication for surgery and alternative CT findings were not retained, so surgery cannot be attributed to the eFAST result. These estimates remain imprecise because of the small verified subgroup and potential verification bias.

### 3.4. Interventions and Mortality

Before-CT tube thoracostomy was recorded in 6/180 (3.3%) pre-protocol patients and 21/180 (11.7%) post-protocol patients (RR 3.50, 95% CI 1.45–8.47; Pearson χ^2^ *p* = 0.003). Blood product use and emergency surgery were not significantly different between phases. ED mortality was 18/180 (10.0%) versus 8/180 (4.4%) (RR 0.44, 95% CI 0.20–1.00; Pearson χ^2^ *p* = 0.042; Fisher’s exact *p* = 0.065). In-hospital mortality was 25/180 (13.9%) versus 14/180 (7.8%) (RR 0.56, 95% CI 0.30–1.04; *p* = 0.062). Among patients surviving the ED episode, subsequent in-hospital deaths numbered seven and six in the pre- and post-protocol groups, respectively. Gunshot wounds were recorded in nine versus four patients; ED deaths among those patients numbered 2/9 and 0/4, respectively. These unadjusted differences are exploratory associations, not evidence that the protocol changed clinical outcomes. Causes and precise times of ED deaths were not available in the analytic dataset (Table 4).

### 3.5. Exploratory Mortality Analysis

The originally submitted multivariable logistic regression model was removed because the patient-level re-analysis did not reproduce the submitted adjusted phase estimate and because only 39 in-hospital deaths occurred. The original model also combined RTS and shock-related measures with overlapping physiologic information. To avoid overfitting and overinterpretation, no adjusted mortality model is presented in the revised main analysis. Crude mortality estimates are retained solely as exploratory secondary outcomes. Injury-severity and physiologic distributions are summarized in Table 5. Available de-identified characteristics of ED deaths are provided in Table S2.

**Table 5:** Injury-severity and physiologic distributions after recalculation of RTS. * Overall chi-square test across ISS bands. † Fisher’s exact test.

Severity Measure	Pre-Protocol (*n* = 180)	Post-Protocol (*n* = 180)	*p*-Value
RTS, mean ± SD	7.39 ± 0.58	7.44 ± 0.52	0.409
RTS, median [IQR]	7.84 [6.90–7.84]	7.84 [6.90–7.84]	0.470
ISS < 16, *n* (%)	93 (51.7)	104 (57.8)	
ISS 16–24, *n* (%)	59 (32.8)	56 (31.1)	0.363 *
ISS ≥ 25, *n* (%)	28 (15.6)	20 (11.1)	
GCS ≤ 8, *n* (%)	3 (1.7)	1 (0.6)	0.623 †
SBP < 90 mmHg, *n* (%)	32 (17.8)	36 (20.0)	0.590
TRISS-predicted mortality, median % [IQR]	1.84 [0.88–4.48]	1.46 [0.87–4.11]	0.412

**Figure 3:**
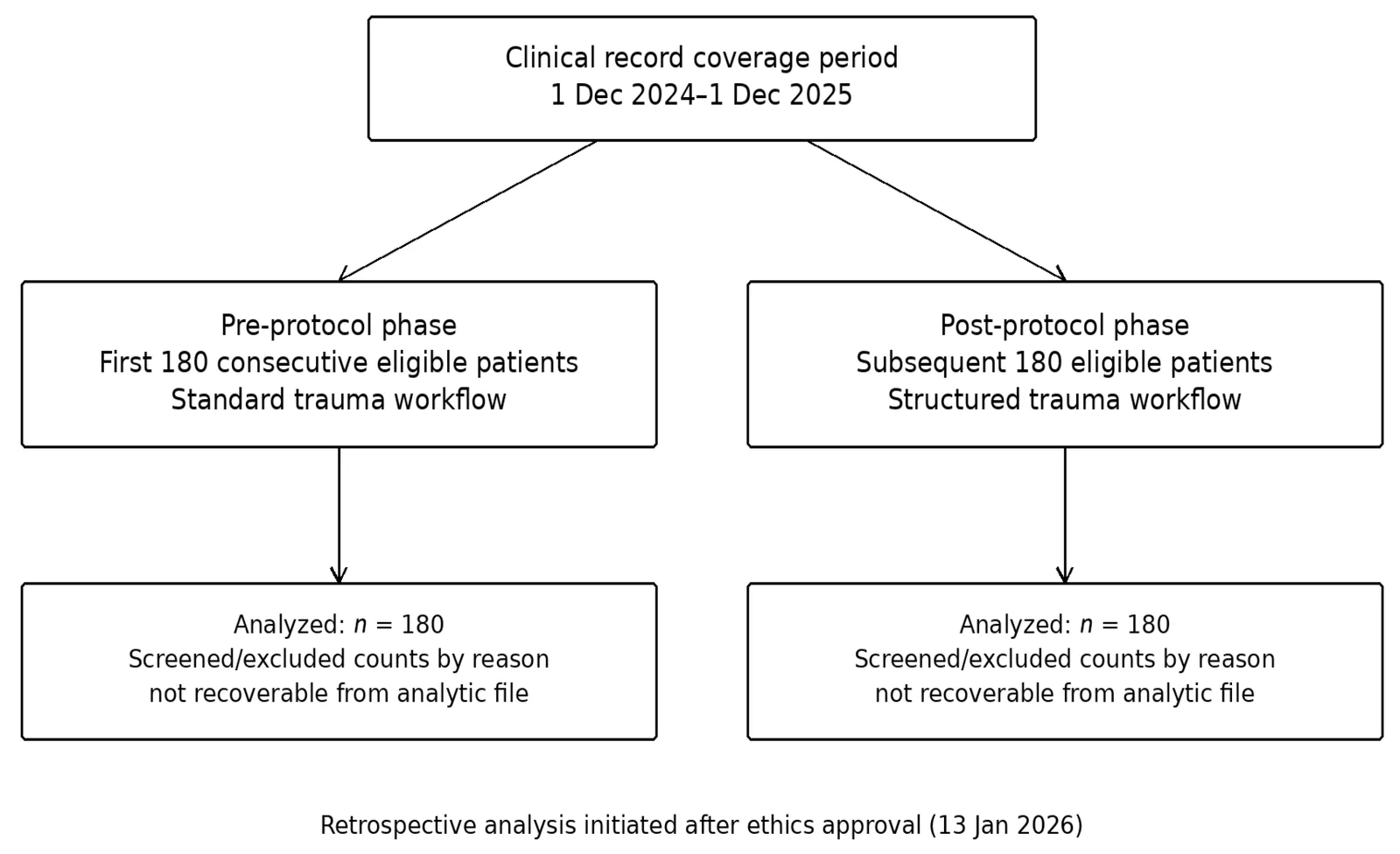
Analytic cohort flow. The de-identified analytic dataset retained the final 180 patients in each phase but not the original screening-log counts by exclusion category; unavailable counts were not estimated.

TRISS was calculated descriptively using the original MTOS coefficients and the recalculated RTS. Median predicted mortality was 1.84% (IQR 0.88–4.48) in the pre-protocol phase and 1.46% (IQR 0.87–4.11) in the post-protocol phase (*p* = 0.412). Because TRISS is a historical prediction model with known calibration limitations across settings and eras, it is used here only to describe case mix and not to compare observed with expected mortality or to support causal inference. The analytic cohort flow is shown in Figure 3.

## 4. Discussion

### 4.1. Principal Findings

CT access, consultation-request, and admission-decision intervals were shorter in the post-protocol phase by approximately 11, 9, and 77 min, respectively, in comparisons of medians. These consistent process-time differences are the study’s main observations. They cannot, however, be assigned causally to the protocol because phase allocation followed calendar time and staffing, patient mix, radiology operations, documentation, and experience may have changed concurrently. Pre-CT thoracostomy was also more frequent in the later phase, but the clinical meaning of this difference is uncertain. Neither the design nor the available data support a conclusion that the workflow improved survival.

The mortality comparisons are especially uncertain. ED mortality differed in the Pearson chi-square analysis, but Fisher’s exact test was not significant, and the crude risk-ratio confidence interval reached approximately 1. In-hospital mortality did not reach the conventional significance threshold. Most of the numerical difference arose during the ED episode (18 vs. 8 deaths); later in-hospital deaths among ED survivors were similar (seven vs. six). Gunshot injuries were more frequent before implementation (nine vs. four). The earlier adjusted mortality estimate could not be reproduced and was removed. Without adjustment for temporal confounding, verification of causes of death, or multiplicity correction, these findings can only generate hypotheses.

### 4.2. Bedside Ultrasound and Early Procedural Care

Early eFAST was intended to make relevant diagnostic information available at the bedside. Thoracic eFAST was documented for all 180 post-protocol patients, but equivalent structured pre-protocol records were unavailable. More pre-CT thoracostomies occurred after implementation; this numerical difference is not necessarily a clinical benefit. Pneumothorax or hemothorax was confirmed in the CT/operative field for 17 of 21 post-protocol pre-CT thoracostomies, while four lacked confirmation in that field. Procedure indications, intent to treat occult pneumothorax, and complications were not retained. The appropriateness and safety of individual procedures therefore cannot be determined from these data.

The abdominal eFAST results also illustrate an important limitation of ultrasound-first strategies. Sensitivity for intra-abdominal free fluid was 66.7% and specificity was 79.2%, with wide confidence intervals in a verified subgroup of only 33 examinations. A negative examination therefore cannot replace definitive imaging in stable patients, and the modest specificity raises the possibility of false-positive bedside findings. Verification bias is possible because reference-standard confirmation was not available for all examinations [9].

### 4.3. CT Coordination and Workflow

The observed difference in CT access time was approximately 50%, but it cannot be attributed solely to CT pre-notification. Documented pre-notification was more common in the post-protocol phase (93.3% vs. 16.7%). Nevertheless, scanner readiness, transfer practices, radiographer availability, reconstruction, PACS workflow, ED crowding, and secular changes may also influence the measured intervals [2,4,12,23,24]. In particular, differences in scan-initiation-to-first-image time cannot be causally linked to the notification step because concurrent scanner, PACS, and staffing information was unavailable.

The shorter consultation-request and admission-decision intervals may have reflected more coordinated clinical activity. Debriefing documentation was more frequent after implementation (81.1% vs. 10.6%), as was CT pre-notification (93.3% vs. 16.7%). These measures support documented uptake of the bundle but cannot isolate any component’s contribution to the observed differences.

### 4.4. Relevance to Resource-Constrained Emergency Care

Hybrid resuscitation–imaging facilities may be unavailable because of cost, space, staffing, or hospital design. Reorganizing existing resources could be feasible in such settings [17,18]. This single-center study describes a pragmatic workflow bundle, but its transferability and independent effects require prospective evaluation. Context and changes over time should be considered when interpreting its findings, consistent with SQUIRE 2.0 [20].

### 4.5. Strengths and Limitations

Strengths include consecutive case selection within each analytic phase, evaluation in the same emergency department, practical workflow measures, and a low-cost bundle that does not require a hybrid imaging facility. The department’s structured electronic record system and routine trauma documentation allowed all four prespecified workflow-time endpoints to be retrieved for every patient in the final 360-patient analytic cohort. This supports the precision of the descriptive workflow comparisons within that cohort, although it cannot control confounding or establish how many earlier records were incomplete or excluded.

This study has important limitations. First, its retrospective, nonrandomized and calendar-sequenced design cannot establish that the protocol caused the observed workflow differences or any clinical outcome. Secular trends, seasonal variation, crowding, trauma volume, staffing, case mix, staff learning, radiology operations, and changes in documentation may have contributed. Second, encounter dates were unavailable in the de-identified analytic file, preventing a monthly run chart, interrupted-time-series analysis, and independent verification of the phase-boundary date. Third, the historical screening log was not retained, so missingness and exclusion counts before formation of the final 360-patient cohort cannot be quantified, despite complete measurement of the four reported workflow-time endpoints within that cohort. Fourth, the bundle’s components cannot be evaluated independently. Fifth, structured pre-protocol eFAST results, operator credentialing, detailed thoracostomy indications and complications, causes and exact timing of ED deaths, and concurrent scanner/PACS/staffing information were unavailable. Sixth, admission-decision intervals were recorded for patients who died in the ED, but their event order could not be independently verified without the original timestamps. Seventh, abdominal eFAST accuracy was estimated in only 33 paired examinations and may be affected by verification bias. Finally, multiple exploratory outcomes were evaluated without multiplicity correction. These limitations constrain interpretation of the clinical outcomes and causal attribution, even though the primary descriptive workflow comparisons can be reproduced from the retained analytic data.

## 5. Conclusions

In this single-center, retrospective before-and-after study, the post-protocol phase was associated with shorter CT access, consultation-request, and admission-decision times. Complete measurement of the four selected workflow intervals in the analytic cohort strengthens their descriptive reporting but does not overcome the uncontrolled, calendar-based design. The observed procedural and mortality differences remain exploratory and cannot establish a causal benefit of the protocol. Prospective controlled studies with temporal and implementation-fidelity measures are required to assess reproducibility and patient-centered effects.

## Figures and Tables

**Table 1:** Baseline characteristics and injury profile.

Variable	Pre-Protocol (*n* = 180)	Post-Protocol (*n* = 180)	*p*-Value	SMD
Age, years, mean ± SD	40.4 ± 15.3	42.5 ± 14.9	0.187	0.139
Male sex, *n* (%)	113 (62.8)	96 (53.3)	0.069	0.191
GCS on arrival, mean ± SD	13.22 ± 1.76	13.55 ± 1.53	0.056	0.202
RTS on arrival, mean ± SD	7.39 ± 0.58	7.44 ± 0.52	0.409	0.087
ISS, mean ± SD	15.77 ± 7.97	14.32 ± 7.27	0.073	0.189
Shock index, mean ± SD	0.78 ± 0.23	0.75 ± 0.21	0.194	0.137
Motor vehicle collision, *n* (%)	63 (35.0)	58 (32.2)	0.577	0.059
Motorcycle/bicycle, *n* (%)	18 (10.0)	22 (12.2)	0.502	0.071
Vehicle–pedestrian, *n* (%)	9 (5.0)	15 (8.3)	0.205	0.134
Falls, *n* (%)	54 (30.0)	58 (32.2)	0.649	0.048
Stab/cut injuries, *n* (%)	18 (10.0)	16 (8.9)	0.719	0.038
Gunshot wounds, *n* (%)	9 (5.0)	4 (2.2)	0.158	0.149
Other mechanism, *n* (%)	9 (5.0)	7 (3.9)	0.609	0.054
Extremity injury, *n*	53	51	0.816	0.025
Head/neck injury, *n*	41	49	0.330	0.103
Thoracic injury, *n*	24	31	0.305	0.108
Abdominal injury, *n*	11	16	0.317	0.105
Pelvic/sacral injury, *n*	9	12	0.500	0.071
Spinal injury, *n*	9	9	1.000	0.000
Polytrauma, *n*	33	41	0.297	0.110

GCS, Glasgow Coma Scale; RTS, Revised Trauma Score; ISS, Injury Severity Score; SD, standard deviation; SMD, standardized mean difference. RTS values were recalculated from recorded GCS, SBP, and respiratory rate using the standard weighted formula. Continuous-variable *p*-values are based on independent-samples comparisons; categorical *p*-values are based on Pearson chi-square tests unless sparse data warranted an exact test. Mechanism-of-injury categories are mutually exclusive. Anatomical injury categories are not mutually exclusive; therefore, anatomical counts are not expected to sum to 180.

**Table 2:** Operational workflow metrics before and after protocol implementation. Values are mean ± SD; median [IQR]. Mean differences are post-protocol minus pre-protocol. *n* = 180 per phase for each workflow metric; no values were missing in the de-identified analytic dataset.

Metric	Pre-Protocol	Post-Protocol	Mean Difference (95% CI)	Mann–Whitney *p*
CT access time, min	22.42 ± 9.55; 22.38 [16.02–28.75]	11.33 ± 5.14; 10.98 [7.53–14.71]	−11.10 (−12.69 to −9.50)	<0.001
Time to first image, min	8.00 ± 3.09; 8.15 [5.75–9.83]	4.02 ± 1.76; 4.01 [2.80–5.22]	−3.99 (−4.51 to −3.46)	<0.001
Consultation-request time, min	23.07 ± 10.22; 23.23 [16.13–30.03]	14.23 ± 6.65; 13.69 [9.68–18.45]	−8.85 (−10.64 to −7.06)	<0.001
Admission-decision time, min	123.41 ± 48.61; 125.95 [88.71–151.98]	49.46 ± 21.12; 49.08 [35.16–64.10]	−73.95 (−81.73 to −66.17)	<0.001

**Table 3:** Diagnostic performance of eFAST for intra-abdominal free fluid.

Metric	Result
True positive	6
False positive	5
True negative	19
False negative	3
Sensitivity	66.7% (95% CI 35.4–87.9)
Specificity	79.2% (95% CI 59.5–90.8)
Positive predictive value	54.5% (95% CI 28.0–78.7)
Negative predictive value	86.4% (95% CI 66.7–95.3)
Accuracy	75.8% (95% CI 59.0–87.2)

Reference standard: CT and/or operative confirmation when available. Confidence intervals are Wilson 95% intervals. The denominator is the 33 examinations with sufficient intra-abdominal reference-standard information.

**Table 4:** Interventions and clinical outcomes.

Outcome	Pre-Protocol *n* (%)	Post-Protocol *n* (%)	Risk Ratio (95% CI)	*p*-Value
Early tube thoracostomy	6 (3.3)	21 (11.7)	3.50 (1.45–8.47)	0.003
Blood product requirement	62 (34.4)	55 (30.6)	0.89 (0.66–1.20)	0.431
Emergency surgery	48 (26.7)	40 (22.2)	0.83 (0.58–1.20)	0.327
ED mortality	18 (10.0)	8 (4.4)	0.44 (0.20–1.00)	0.042
In-hospital mortality	25 (13.9)	14 (7.8)	0.56 (0.30–1.04)	0.062

## Data Availability

The patient-level analytic dataset is not publicly available because of institutional and ethical data-sharing restrictions. De-identified data supporting the findings of this study may be made available by the corresponding author upon reasonable request and subject to institutional and ethical approval.

## Supplementary Materials

The following supporting information can be downloaded at: https://www.mdpi.com/article/10.3390/healthcare14213588/s1, Table S1, Operational definitions and decision bundle; Table S2, De-identified characteristics of patients who died in the ED; Figure S1, Structured trauma workflow.
