# Supplementary material for: Workflow and Clinical Outcomes After Implementation of a Structured Trauma Protocol in a Resource-Constrained Emergency Department: A Single-Center Retrospective Before-and-After Cohort Study
Source: Healthcare (Basel). 2026 Oct 23;14(21):3588. doi: 10.3390/healthcare14213588 (PMC13763155; doi:10.3390/healthcare14213588)
Supplement: Supplementary file 1 [file healthcare-14-03588-s001.zip › Supplementary_Table_S1.pdf]

Supplementary Table S1. Operational definitions and decision bundle

Manuscript ID: healthcare-4606753

Component	Trigger / timing	Operational action	Fidelity / limitation
Trauma-team activation	Existing departmental trauma-team activation; exact historical threshold values were not retained in the de-identified analytic file.	Trauma-team evaluation initiated; routine initial resuscitation proceeded.	No phase-specific activation-threshold change could be independently re-verified from the analytic file.
Early eFAST	Post-protocol: during initial resuscitation.	Pericardial, intraperitoneal, pleural and lung views incorporated into bedside assessment.	Thoracic eFAST result retained for 180/180 post-protocol patients; structured pre-protocol eFAST fields were not retained.
Immediate pre-CT intervention	If the treating team judged a thoracic injury to require immediate treatment before CT.	Tube thoracostomy could be performed before CT.	6/180 pre-protocol and 21/180 post-protocol; CT/operative PTX/HTX confirmation present in 17/21 post-protocol cases. Detailed indications and complications were not retained.
CT prioritization	At trauma-team activation or immediately on ambulance arrival in the structured workflow.	CT personnel pre-notified; scanner readiness and patient transfer coordinated in parallel with stabilization.	Documented pre-notification: 30/180 (16.7%) pre vs 168/180 (93.3%) post.
Consultation	After initial assessment/stabilization and according to identified injuries.	Specialty consultation request recorded as elapsed time from ED arrival.	Exact historical specialty-specific trigger thresholds were not retained.
Disposition decision	Following initial resuscitation, diagnostic evaluation and specialty input.	Admission/disposition decision interval recorded from ED arrival.	An interval was present for all 360 analytic records; raw event timestamps were not retained.
Micro-debriefing	After major trauma resuscitation when feasible.	Brief team-leader review of delays, communication, role allocation, equipment/spatial constraints and practical changes.	Documented debriefing: 19/180 (10.6%) pre vs 146/180 (81.1%) post.
