# Supplementary material for: Workflow and Clinical Outcomes After Implementation of a Structured Trauma Protocol in a Resource-Constrained Emergency Department: A Single-Center Retrospective Before-and-After Cohort Study
Source: Healthcare (Basel). 2026 Oct 23;14(21):3588. doi: 10.3390/healthcare14213588 (PMC13763155; doi:10.3390/healthcare14213588)
Supplement: Supplementary file 1 [file healthcare-14-03588-s001.zip › Supplementary_Table_S2.pdf]

Supplementary Table S2. De-identified characteristics of patients who died in the ED

Manuscript ID: healthcare-4606753

Study ID	Phase	Mechanism	GCS	SBP	RTS	ISS	TRISS predicted mortality (%)	Cause / exact timing
T015	Pre-protocol	Motorcycle/bicycle	11	125	6.904	22	3.57	Not retained in de-identified analytic file
T027	Pre-protocol	Fall	8	126	5.9672	32	51.01	Not retained in de-identified analytic file
T036	Pre-protocol	Motor vehicle collision	12	91	6.904	21	3.3	Not retained in de-identified analytic file
T047	Pre-protocol	Motor vehicle collision	9	81	6.1714	29	40.73	Not retained in de-identified analytic file
T061	Pre-protocol	Gunshot	9	109	6.904	35	11.46	Not retained in de-identified analytic file
T066	Pre-protocol	Fall	7	82	5.2346	33	26.37	Not retained in de-identified analytic file
T079	Pre-protocol	Motor vehicle collision	11	87	6.1714	29	10.73	Not retained in de-identified analytic file
T081	Pre-protocol	Motor vehicle collision	12	129	6.904	22	3.57	Not retained in de-identified analytic file
T108	Pre-protocol	Stab/cut	9	124	6.904	30	8.55	Not retained in de-identified analytic file
T116	Pre-protocol	Gunshot	12	98	6.904	29	8.05	Not retained in de-identified analytic file
T120	Pre-protocol	Motor vehicle collision	11	91	6.904	19	14.15	Not retained in de-identified analytic file
T131	Pre-protocol	Fall	8	101	5.9672	34	17.72	Not retained in de-identified analytic file
T136	Pre-protocol	Other	15	113	7.8408	11	0.69	Not retained in de-identified analytic file
T139	Pre-protocol	Motor vehicle collision	10	111	6.904	24	4.2	Not retained in de-identified analytic file
T144	Pre-protocol	Motor vehicle collision	10	114	6.904	31	7.29	Not retained in de-identified analytic file
T158	Pre-protocol	Motor vehicle collision	11	130	6.904	21	3.3	Not retained in de-identified analytic file
T170	Pre-protocol	Fall	12	97	6.904	18	13.17	Not retained in de-identified analytic file
T180	Pre-protocol	Fall	12	84	6.1714	28	9.96	Not retained in de-identified analytic file
T182	Post-protocol	Vehicle-pedestrian	12	126	6.904	23	3.87	Not retained in de-identified analytic file
T184	Post-protocol	Fall	10	102	6.904	28	5.77	Not retained in de-identified analytic file
T190	Post-protocol	Vehicle-pedestrian	13	91	7.8408	18	1.23	Not retained in de-identified analytic file
T204	Post-protocol	Fall	12	124	6.904	25	21.39	Not retained in de-identified analytic file
T244	Post-protocol	Fall	11	124	6.904	32	7.87	Not retained in de-identified analytic file
T264	Post-protocol	Stab/cut	11	102	6.904	20	4.65	Not retained in de-identified analytic file
T317	Post-protocol	Fall	8	126	5.9672	29	12.42	Not retained in de-identified analytic file
T320	Post-protocol	Motor vehicle collision	9	117	6.904	26	4.92	Not retained in de-identified analytic file

Note: All ED deaths were also classified as in-hospital deaths. Cause and exact timing of death were not retained in the de-identified analytic dataset. Table S2 is provided to show the available case-mix and physiologic information without inventing unavailable adjudication details.
